# Electrocatalytic Reduction of Nitrate *via* Co_3_O_4_/Ti Cathode Prepared by Electrodeposition Paired With IrO_2_-RuO_2_ Anode

**DOI:** 10.3389/fchem.2022.900962

**Published:** 2022-06-03

**Authors:** Chuan Wang, Zhifen Cao, Hongtao Huang, Hong Liu, Sha Wang

**Affiliations:** ^1^ Key Laboratory for Water Quality and Conservation of the Pearl River Delta, Ministry of Education, Institute of Environmental Research at Greater Bay, Guangzhou University, Guangzhou, China; ^2^ Chongqing Institute of Green and Intelligent Technology, Chinese Academy of Sciences, Chongqing, China

**Keywords:** nitrate removal, electrocatalytic, Co_3_O_4_/Ti, reduction, IrO_2_-RuO_2_

## Abstract

Nitrate pollution is already a global problem, and the electrocatalytic reduction of nitrate is a promising technology for the remediation of wastewater and polluted water bodies. In this work, Co_3_O_4_/Ti electrodes were prepared by electrodeposition for the electrocatalytic reduction of nitrate. The morphology, chemical, and crystal structures of Co_3_O_4_/Ti and its catalytic activity were investigated. Then, the electrocatalytic nitrate reduction performance of Co_3_O_4_/Ti as the cathode was evaluated by monitoring the removal efficiencies of nitrate (NO_3_^−^-N) and total nitrogen (TN), generation of reduction products, current efficiency (CE), and energy consumption (EC) at different operating conditions. Under the catalysis of Co_3_O_4_/Ti, NO_3_^−^ was reduced to N_2_ and NH_4_^+^, while no NO_2_^−^ was produced. After the introduction of chloride ions and IrO_2_-RuO_2_/Ti as the anode, NH_4_^+^ was selectively oxidized to N_2_. The removal efficiencies of NO_3_^−^-N (at 100 mg/L) and TN after 2 h were 91.12% and 60.25%, respectively (pH 7.0; Cl^−^ concentration, 2000 mg/L; current density, 15 mA/cm^2^). After 4 h of operation, NO_3_^−^-N and TN were completely removed. However, considering the EC and CE, a 2-h reaction was the most appropriate. The EC and CE were 0.10 kWh/g NO_3_^−^N and 40.3%, respectively, and electrocatalytic performance was maintained after 10 consecutive reduction cycles (2 h each). The cathode Co_3_O_4_/Ti, which is prepared by electrodeposition, can effectively remove NO_3_^−^-N, with low EC and high CE.

## 1 Introduction

Nitrate (NO_3_^−^) contamination of surface water and groundwater is a global environmental problem associated with increasing populations, and its hazards have attracted much attention (Jasper et al., 2014; Khalil et al., 2016; Serio et al., 2018). The accumulation of plant nutrients such as NO_3_^−^ and phosphate in water can accelerate eutrophication, a process that increases the biomass of a water body as its biological diversity decreases, for example, due to increases in invertebrates and fish. In the extreme, a state of hypoxia can exist, resulting in the loss of the aquatic ecosystems (Kubicz et al., 2018; Zhang et al., 2021). Although NO_3_^−^ is chemically stable, it can be microbially reduced to reactive nitrite in the oral cavity and stomach, which has been linked to liver damage, methemoglobinemia, and cancer in animals (Spalding and Exner, 1993; Elmidaoui et al., 2001; Barakat et al., 2020).

Currently, microbial denitrification is widely used for the large-scale remediation of NO_3_^−^ pollution (Clauwaert et al., 2007; Della Rocca and Belgiorno V Meriç, 2007). Many other methods of NO_3_^−^ removal have been explored such as reverse osmosis, ion exchange, ammonia stripping, electrodialysis, catalytic reduction, and electrocatalytic reduction (Kapoor and Viraraghavan, 1997; Yang and Lee, 2005; Della Rocca and Belgiorno V Meriç, 2007). Among these techniques, the electrocatalytic reduction of NO_3_^−^ is a promising and clean technology because the electron reductants neither introduce pollutants nor adversely affect the environment (Garcia-Segura et al., 2018; Gayen et al., 2018).

The mechanism of the electrochemical NO_3_^−^ reduction reaction (NO_3_^−^-RR) involves anodic oxidation and cathodic reduction in which NO_3_^−^ is reduced to NO_2_^−^, NH_4_^+^, and N_2_ on the active sites of the cathode according to Eqs 1–3 (Zhang et al., 2021):

$$NO_{3}^{-}+2e^{-}+H_{2}O\to NO_{2}^{-}+2OH^{-},$$ (1)

$$NO_{2}^{-}+6e^{-}+6H_{2}O\to NH_{4}^{+}+8OH^{-},$$ (2)

$$2NO_{3}^{-}+10e^{-}+6H_{2}O\to N_{2}+12OH^{-}.$$ (3)

The choice of the cathode material is important in this process. To date, most studies have used high-cost noble metal cathodes, such as Pt, Rh, and Pd, which may limit their commercial application (Taguchi and Feliu, 2007; Yang et al., 2014; Soto-Hernández et al., 2019). Co_3_O_4_ is a cost-effective catalyst, and the preparation of a CuO-Co_3_O_4_/Ti electrode by the sol-gel method for electrochemical reduction of NO_3_^−^ was recently reported (Yang et al., 2020). The system demonstrated the complete removal of NO_3_^−^ after 3 h at a current density of 20 mA/cm^2^.

NO_2_^−^ and NH_4_^+^ generated at the cathode (Eqs. (1) and (2)) diffuse to the anode where they are adsorbed onto the surface and subsequently oxidized to NO_3_^−^ and N_2_ (Eqs. (4) and (5)) (Zhang et al., 2021):

$$NO_{2}^{-}+H_{2}O\to NO_{3}^{-}+2e^{-}+2H^{+},$$ (4)

$$2NH_{4}^{+}\to N_{2}+6e^{-}+6H^{+}.$$ (5)

When Cl^−^ is present in the electrolyte, the following reactions also occur at the anode (Eqs. 6–9) (Zhang et al., 2021):

$$2Cl^{-}\to Cl_{2}+2e^{-},$$ (6)

$$Cl_{2}+H_{2}O\to HOCl+Cl^{-}+H^{+},$$ (7)

$$HOCl\to ClO^{-}+H^{+},$$ (8)

$$2NH_{4}^{+}+3ClO^{-}\to N_{2}+3H_{2}O+2H^{+}+3Cl^{-}.$$ (9)

The electrochemical NO_3_^−^-RR involves NO_3_^−^ reduction at the cathode and ammonium nitrogen (NH_4_^+^-N) oxidation at the anode. Cl_2_ generated at the anode (Eq. (6)) immediately forms hypochlorite (Eq. (7)), which selectively oxidizes NH_4_^+^ to N_2_ (Su et al., 2017). Hence, the efficient anodic oxidation of chloride ions is a key requirement for this process, and the anode materials used in the chlor-alkali industry, which obtain Cl_2_ by electrolysis of sodium chloride, provide a useful reference (Yi et al., 2007). Among these materials, IrO_2_-RuO_2_ is a good choice due to its low overpotential, high chlorine selectivity, and long-term stability (Chen et al., 2007). In addition, the electrocatalytic reduction of NO_3_^−^-N is also affected by reaction potential, current, solution pH, battery structure, and anode material.

Here, a catalytic cathode was prepared by the *in situ* electrodeposition of Co_3_O_4_ on a titanium substrate (Co_3_O_4_/Ti) to obtain improved electrocatalytic performance. IrO_2_-RuO_2_/Ti was employed as the anode for the effective removal of NH_4_^+^-N and TN. The aim of this study was to obtain simultaneous electrochemical NO_3_^−^ reduction and oxidation of the *in situ*-generated NO_2_^−^ and NH_4_^+^ into N_2_ gas. The morphology and structure of Co_3_O_4_/Ti were characterized using conventional methods, and its performance in NO_3_^−^ removal was evaluated under different operating conditions. The current efficiency (CE) and energy consumption (EC) of the system were also measured to assess its commercial application.

## 2 Experimental Section

### 2.1 Chemicals and Materials

The Ti mesh and Ti plate (99.5% purity, 0.6 mm, 10 mesh) were purchased from Lanruiyinde Electrochemical Materials Co., Ltd. (China). The Pt plate was obtained from Aidahengsheng Co., Ltd., (Tianjin, China). All chemicals were of analytical grade. Potassium nitrate and sodium hydroxide were purchased from Aladdin Biochemical Technology Co., Ltd. (Shanghai, China). Cobalt nitrate hexahydrate, sodium eicosyl, hexachloroiridic acid, and ruthenium (III) chloride were obtained from Macklin Biochemical Technology Co., Ltd. (Shanghai, China). Solutions were prepared using deionized water (>15 MΩ cm) obtained from an Elix^®^ 3 purification system (Millipore, United States). Simulated wastewater was prepared by adding potassium nitrate to deionized water.

### 2.2 Preparation of Co_3_O_4_/Ti Cathode and IrO_2_-RuO_2_/Ti Anode

Samples of the Ti mesh and Ti plate (3 × 4 cm, 12 cm^2^) were degreased with NaOH solution (40 wt%) at 95°C for 2 h before etching by boiling in oxalic acid solution (10 wt%) for 2 h. The treated samples were then rinsed with deionized water and stored in ethanol until further use.

**FIGURE 1:**
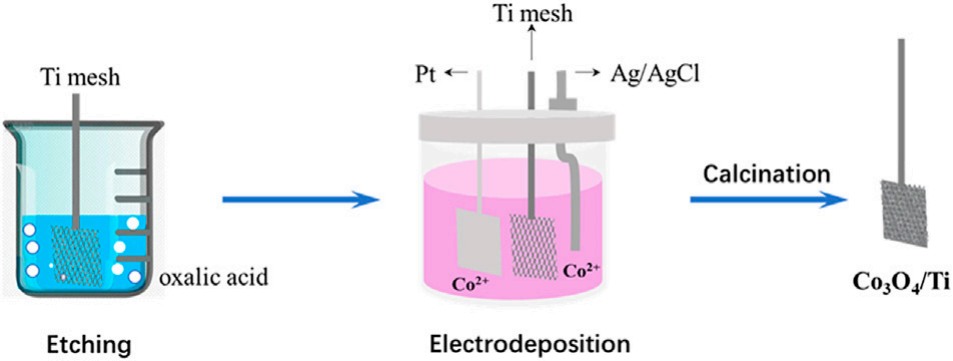
Schematic of the preparation procedure.

As shown in Figure 1, the Co_3_O_4_/Ti electrode was prepared using an electrodeposition method. A three-electrode system was employed in a single compartment cell using the pretreated Ti mesh as the cathode, the Pt plate as the anode, and an Ag/AgCl reference electrode. The electrodeposition solution comprised boric acid (0.5 M), cobalt nitrate hexahydrate (0.1 M), and sodium eicosyl sulfonate (2.0 g/L). Following electrodeposition at a current of 0.25 A for 5 min, the electrode was cleaned with deionized water and oven-dried (60°C) before heating at 5°C/min to 500°C (hold 2 h) in a muffle furnace to effect calcination. The treated samples were allowed to cool naturally to room temperature.

The IrO_2_-RuO_2_/Ti anode was prepared by using a thermal decomposition method. A mixed solution of hexachloroiridic acid and ruthenium (III) chloride in n-butanol (molar ratio, 2:1) was evenly coated onto the surfaces of the pretreated titanium plate, dried at 105°C for 10 min, and then calcined at 500°C for 15 min. The process was repeated until the weight of the titanium plate increased by about 10 g/cm^2^. Finally, the electrode was washed with deionized water before use.

### 2.3 Characterization of the Co_3_O_4_/Ti Cathode

Surface morphology and elemental composition were studied by field-emission scanning electron microscopy (SEM) and energy-dispersive X-ray spectroscopy (EDS) on a Phenom ProX system (Thermo Fisher Scientific, United States) at an accelerating voltage of 15 kV. The crystal structure of Co_3_O_4_ was examined by X-ray diffraction (XRD) with an X’pert Powder system (Malvern Panalytical, Malvern, UK) using Cu Kα (*λ* = 1.5406 Å) irradiation.

### 2.4 Electrochemical Measurements

NO_3_^−^-RR tests were performed in a single chamber electrolytic cell (200 ml) using a three-electrode system, with Co_3_O_4_/Ti (or Ti as required), Pt plate, and Ag/AgCl as the working, counter, and reference electrodes, respectively. The electrolyte was prepared using Na_2_SO_4_ (0.1 M) and different concentrations of NO_3_^−^-N (KNO_3_) as required. Linear sweep voltammetry (LSV), and electrolysis tests were performed in an electrochemical workstation (Metrohm Autolab M204, Switzerland). Prior to the electrochemical test, oxygen was removed by bubbling high-purity N_2_ through the electrolyte for ≥20 min, and continuously fed during the experiments.

Electrolysis measurements were performed at an optimum current density of 15 mA/cm^2^, and aliquots of the reaction solutions (2 ml) were removed at predetermined time intervals to measure the concentrations of NO_3_^−^-N, NO_2_^−^-N, and NH_4_^+^-N. The effects of chlorine on NO_3_^−^-RR and the stability of the Co_3_O_4_/Ti cathode electrode were assessed at a current density of 15 mA/cm^2^ for 2 h and an initial NO_3_^−^-N concentration of 100 mg/L.

### 2.5 Analytical Methods

During the NO_3_^−^-RR, the formation of NO, N_2_O, and NH_3_ are negligible, and hence, the generated gaseous products can be considered as N_2_ (Teng et al., 2018). UV-Vis spectroscopy was used to measure the concentrations of NO_3_^−^-N, NO_2_^−^-N, NH_4_^+^-N, and total nitrogen (TN) (Evolution 201, Thermofisher Scientific Co., Ltd.), and their removal efficiencies were calculated according to Eqs (10)–(12):

$$NO_{3}^{-}-N\;removal=\frac{C_{0}\left(NO_{3}^{-}-N\right)-C_{t}\left(NO_{3}^{-}-N\right)}{C_{0}\left(NO_{3}^{-}-N\right)}\times 100\%,$$ (10)

$$NH_{4}^{+}-N\;generation=\frac{C_{t}\left(NH_{4}^{+}-N\right)}{C_{0}\left(NO_{3}^{-}-N\right)}\times 100\%,$$ (11)

$$TN\;removal=\frac{C_{0}\left(TN\right)-C_{t}\left(TN\right)}{C_{0}\left(TN\right)}\times 100\%,$$ (12)

where C_0_(NO_3_^−^-N) (mg/L) is the initial concentration of NO_3_^−^-N, C_t_(NO_3_^−^-N) (mg/L) is the concentration of NO_3_^−^-N at time t, C_t_(NH_4_^+^-N) (mg/L) is the concentration of NH_4_^+^-N at time t, C_0_(TN) (mg/L) is the initial concentration of TN, and C_t_(TN) (mg/L) is the concentration of TN at time t.

EC was calculated using Eq. 13 (Zhang et al., 2016):

$$EC=\frac{UIt}{V\left(C_{0}-C_{t}\right)},$$ (13)

where *U* is the cell potential (V), I is the current (A), t is the reaction time (h), and V is the volume of reaction solution (L).

The CE for TN removal rates was obtained using Eq. 14:

$$CE\left(\%\right)=\frac{\left(C_{0}-C_{t}\right)\times V}{M\times Q}\times n\times 96485\times 100\%,$$ (14)

where *M* is the molar mass of N (14 g/mol), *Q* is the amount of electricity passing through the electrode, and *n* is the number of electrons obtained from the complete reduction of NO_3_^−^-N (calculated according to the conversion of NO_3_^−^ to N, n = 5).

## 3 Results and Discussion

### 3.1 Electrode Characterizations and Chemical Tests

**FIGURE 2:**
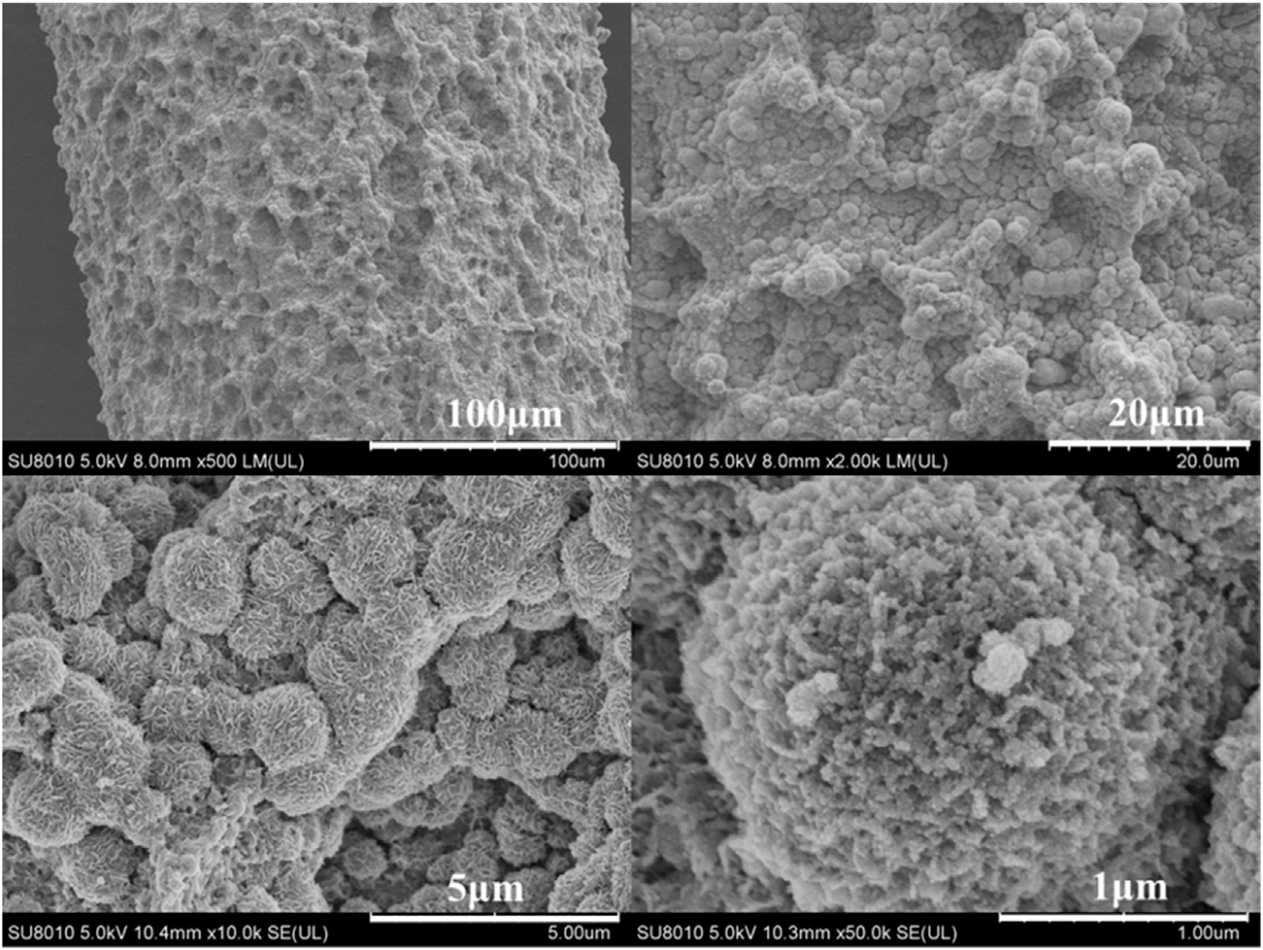
SEM image of Co_3_O_4_/Ti at different resolutions.

**FIGURE 3:**
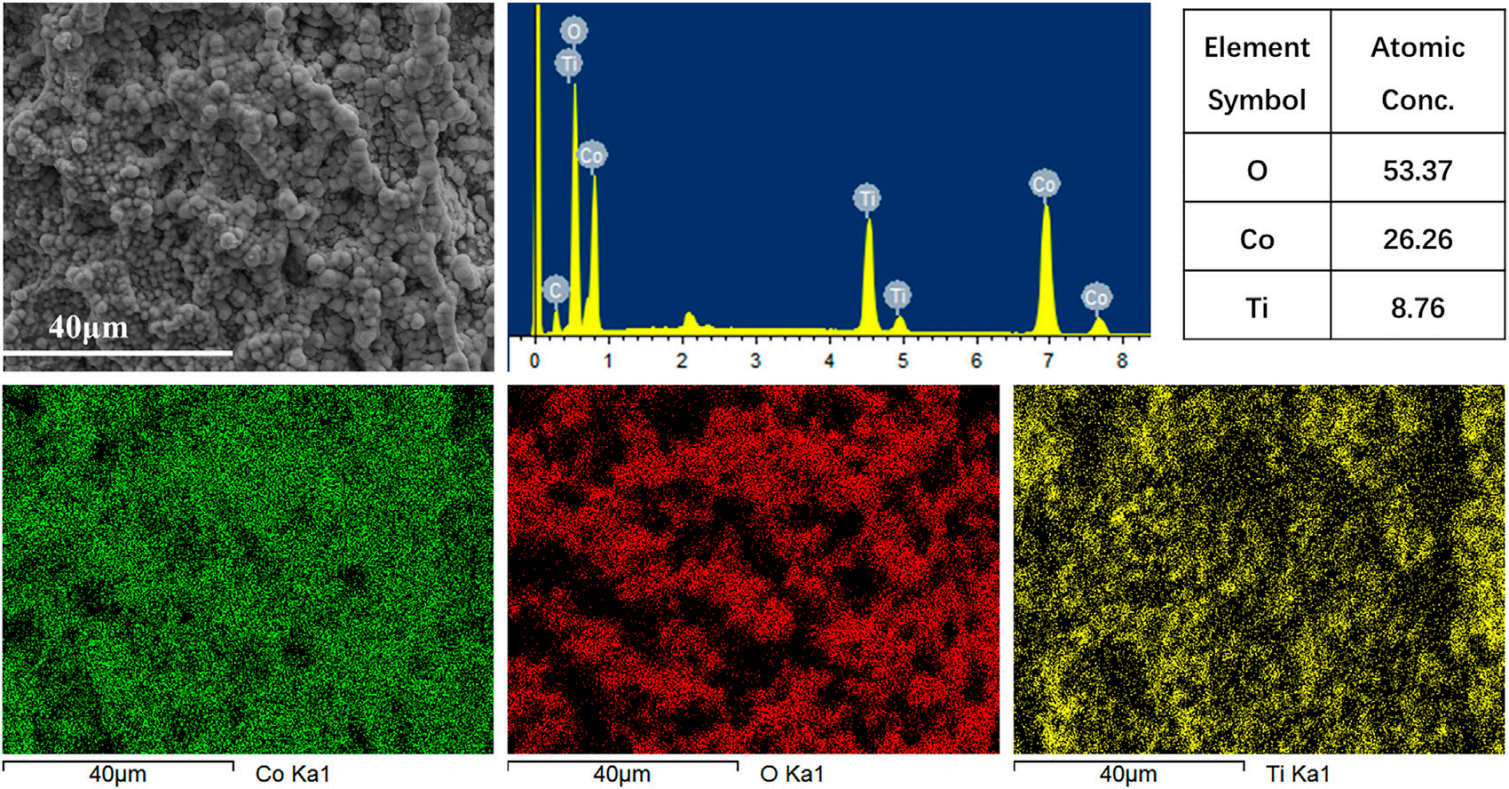
EDS elemental analysis of the surface of Co_3_O_4_/Ti cathode.

SEM was used to depict the electrode surface morphology of Co_3_O_4_/Ti. Figure 2 shows that spherical particles (3–5 μm) of Co_3_O_4_ were observed on the surface of Co_3_O_4_/Ti at different magnifications, confirming its deposition on the Ti mesh. SEM-EDS elemental mapping of a surface region of the Co_3_O_4_/Ti cathode (Figure 3) gave a value of 26.26 atom% for Co, indicating that the element was successfully deposited on the titanium mesh.

**FIGURE 4:**
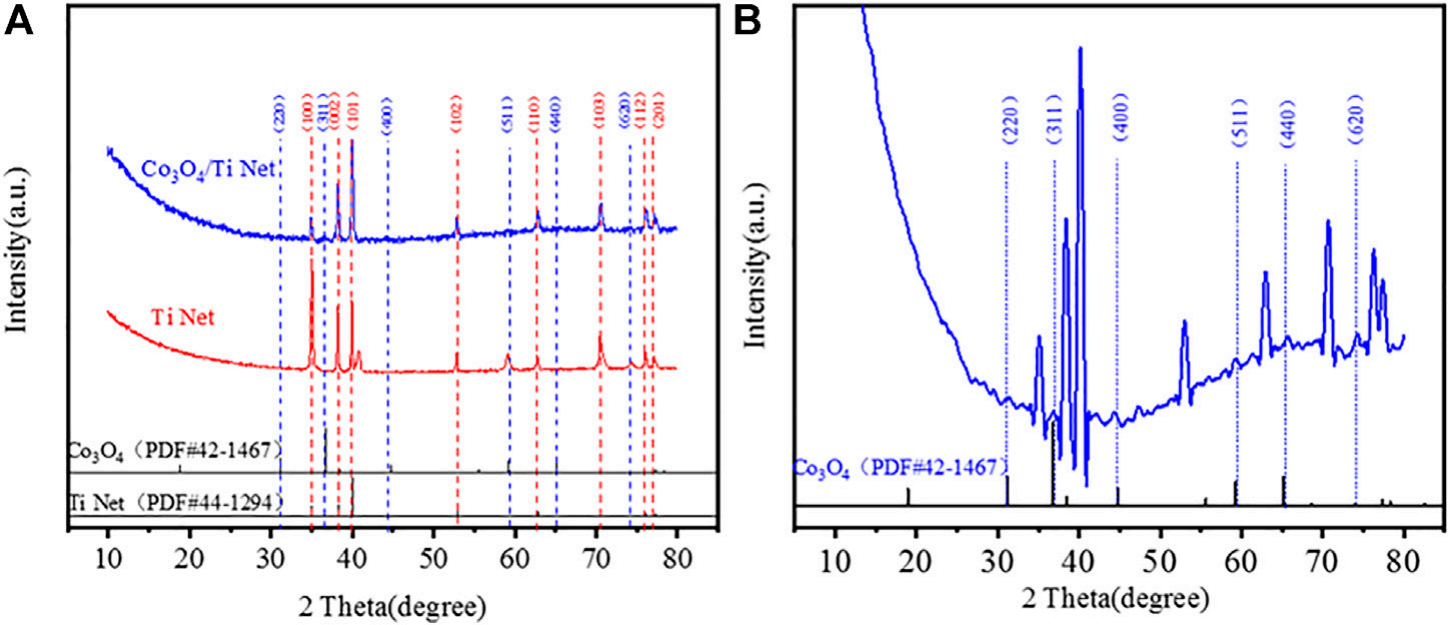
Crystal structure of Co_3_O_4_/Ti cathode and comparison with the reference XRD powder patterns: **(A)** XRD patterns of Ti and Co_3_O_4_/Ti cathodes; **(B)** enlarged XRD patterns of Co_3_O_4_/Ti cathode.

Figure 4 shows the XRD patterns of the calcined Co_3_O_4_/Ti electrode and their comparison with the reference powder patterns of cubic phase Co_3_O_4_ (PDF#42–1467) and Ti (PDF#44–1294). The characteristic peaks observed at 2θ of 35.1°, 38.4°, 40.2°, 53.0°, 62.9°, 70.7°, 76.2°, and 77.4° correspond to (100), (002), (101), (102), (110), (103), (112), and (201) planes of Ti (PDF#44–1294), respectively (Figure 4A). Inspection of the enlarged pattern obtained from the Co_3_O_4_/Ti cathode (Figure 4B) showed that the main peaks of Co_3_O_4_ at 2θ=31.3°, 36.9°, 44.8°, 59.4°, 65.2°, and 74.1°, correspond to the (220), (311), (400), (511), (440), and (620) planes of Co_3_O_4_, respectively. These results were in good agreement with the standard cubic phase (PDF#42–1467).

**FIGURE 5:**
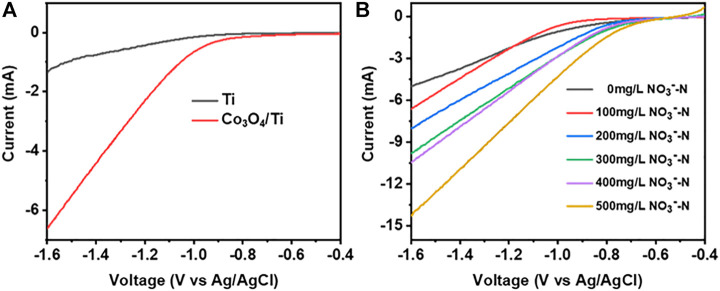
LSV curves illustrating the NO_3_^−^-N removal performance of the catalysts (0.1 M Na_2_SO_4_, 10 mV/s). **(A)** Effects of different catalysts at constant initial NO_3_^−^-N concentration (100 mg/L). **(B)** Effects of increasing initial NO_3_^−^-N concentrations using the Co_3_O_4_/Ti cathode.

LSV was used to evaluate the electrocatalytic performance of the catalysts toward NO_3_^−^-RR. Figure 5A shows the LSV curves obtained with Co_3_O_4_/Ti and Ti in the presence of NO_3_^−^-N. The onset potential for NO_3_^−^-RR using the Co_3_O_4_/Ti cathode (-0.7 V) was more positive than that using the Ti mesh (-1.0 V), indicating the improved performance with the composite catalyst. From −0.7 to −1.6 V, Co_3_O_4_/Ti gave a larger current response at all potentials due to its higher activity toward the NO_3_^−^-RR compared with the Ti mesh. Figure 5B shows the effects of increasing NO_3_^−^-N (0–500 mg/L) using Co_3_O_4_/Ti as the cathode. In the absence of NO_3_^−^-N, the onset potential (i.e., for the electrolysis of water to produce H_2_) was −0.9 V. The addition of NO_3_^−^ produced a positive shift in the onset potential, and the corresponding current increased with increasing initial NO_3_^−^-N due to the enhanced reduction reaction activity.

### 3.2 Effects of Electrochemical Reaction Parameters on NO_3_^−^-RR Using the Co_3_O_4_/Ti Cathode

#### 3.2.1 Catalytic Activity of the Co_3_O_4_/Ti Cathode

**FIGURE 6:**
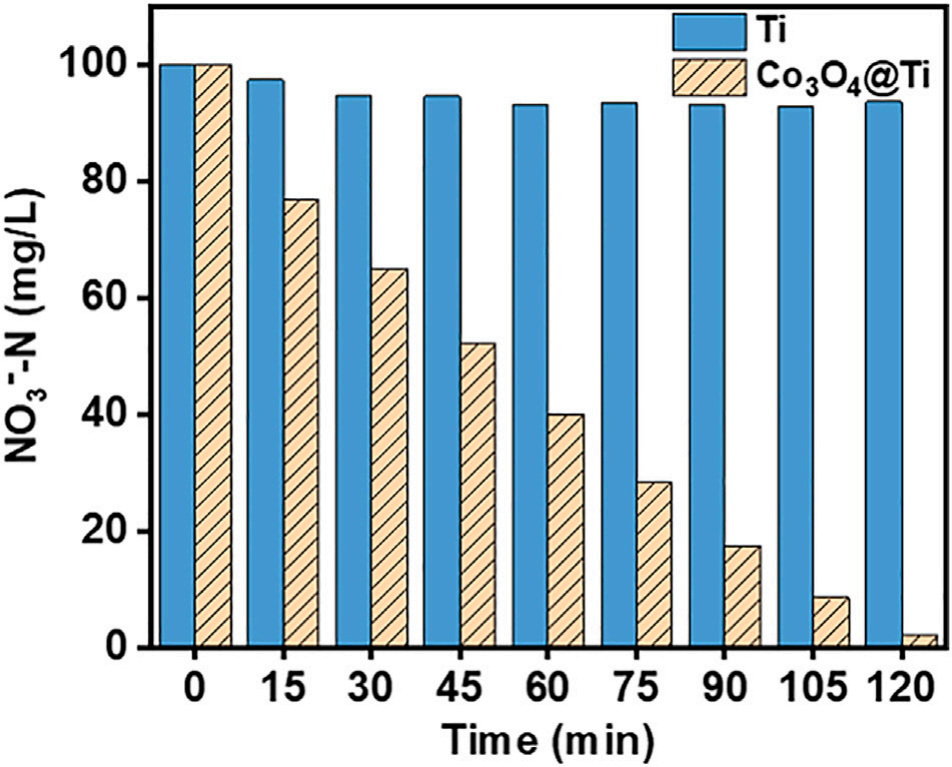
Removal efficiencies of NO_3_^−^-N by the Ti and Co_3_O_4_/Ti cathodes (Pt anode; [NO_3_^−^-N], 100 mg/L, 150 ml; current density, 15 mA/cm^2^; pH, 7.0; reaction time, 2 h).

To determine the effect of Co_3_O_4_ on NO_3_^−^RR activity, the NO_3_^−^-N (100 mg/L) removal efficiencies of the Co_3_O_4_/Ti and Ti mesh cathodes were compared at a current density of 15 mA/cm^2^ with a Pt plate as the anode (Figure 6). The results showed that the Co_3_O_4_/Ti cathode could achieve a NO_3_^−^-N removal efficiency of ∼98% in 2 h, compared with 6.4% using the Ti mesh, demonstrating the important role of Co_3_O_4_ in improving the performance of NO_3_^−^-RR.

#### 3.2.2 Effects of Current Density

**FIGURE 7:**
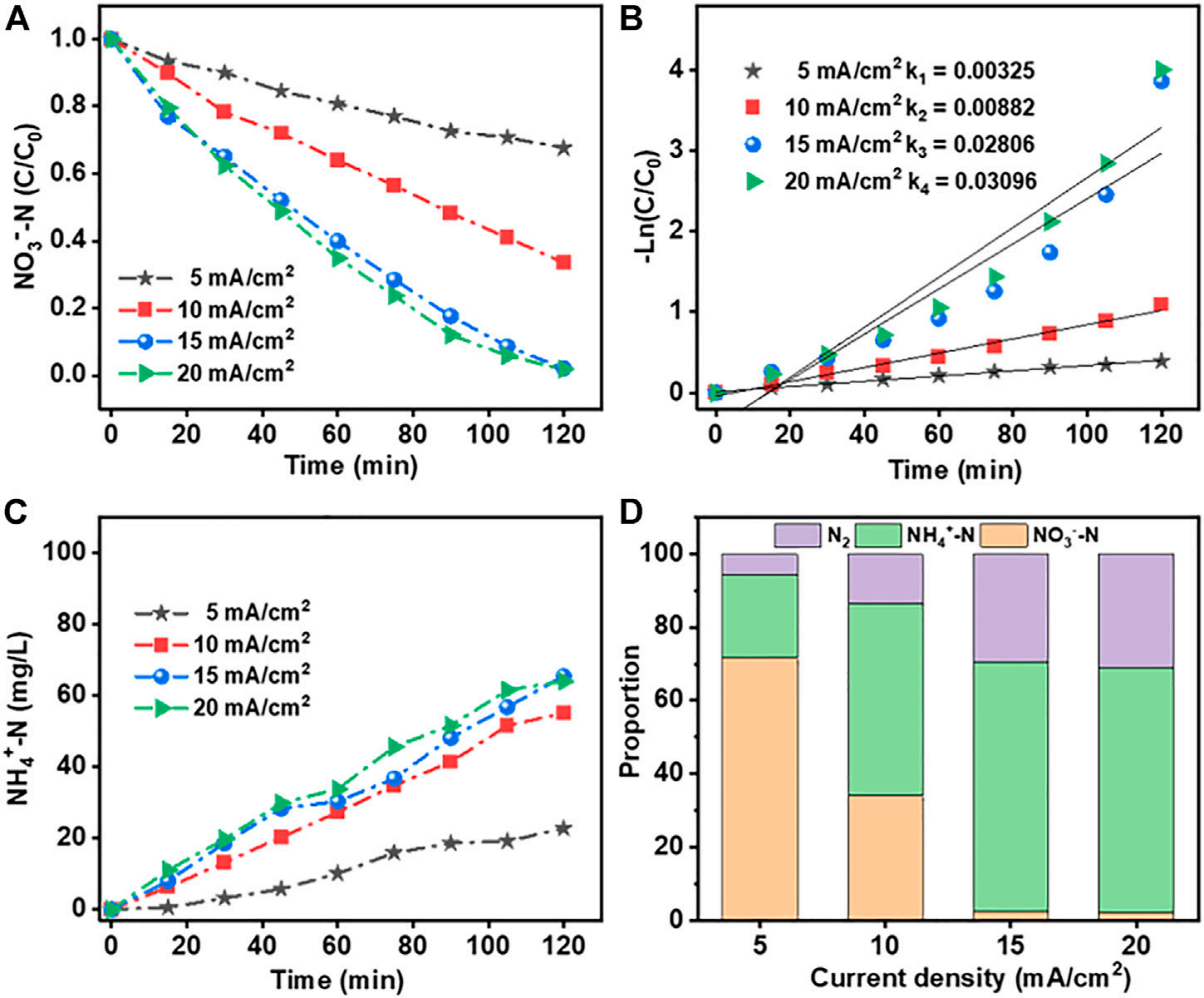
Effects of current density on the NO_3_^−^-RR using the Co_3_O_4_/Ti cathode (Pt anode; [NO_3_^−^-N], 100 mg/L, 150 ml; initial pH, 7.0; reaction time, 2 h): **(A)** and **(B)** rate of loss of NO_3_^−^-N and the fit of the data to first-order kinetics; **(C)** rate of generation of NH_4_^+^-N; and **(D)** distribution of the products formed.

Figure 7A,B show the rate of NO_3_^−^-N removal using the Co_3_O_4_/Ti cathode and the corresponding fit of the experimental data to first-order kinetics. The increased removal efficiency with increasing current density over 5–15 mA/cm^2^ could be attributed to enhanced electron transfer on the electrode surface of Co_3_O_4_/Ti, which increased the rate of NO_3_^−^-RR. However, when the current density was increased from 15 to 20 mA/cm^2^, the removal efficiency of NO_3_^−^-N did not improve significantly. At higher current densities, the competing hydrogen evolution reaction consumes the extra charge, and the NO_3_^−^-N removal efficiency decreases. Figure 7C shows that there was good correspondence between the reduction of NO_3_^−^-N and the generation of NH_4_^+^-N. The reduction products were NH_4_^+^-N and N_2_, while NO_2_^−^-N was not detected (Figure 7D).

#### 3.2.3 Effect of Initial NO_3_^−^-N Concentration

**FIGURE 8:**
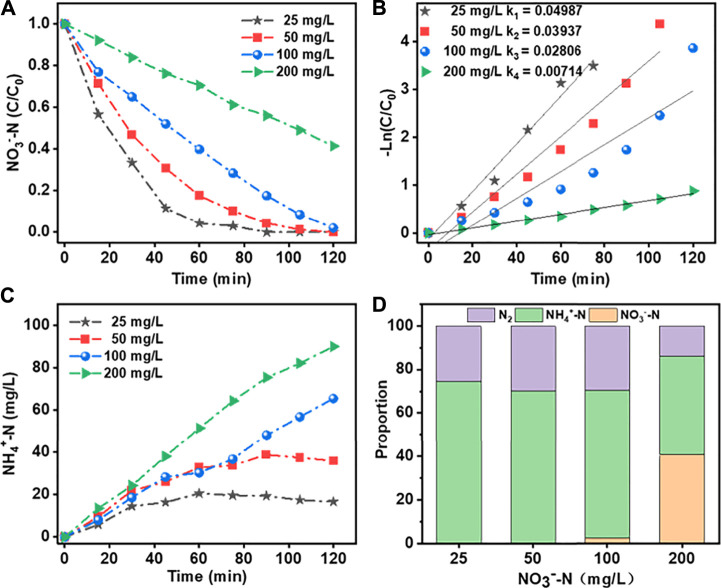
Effects of different initial NO_3_^−^-N concentrations on NO_3_^−^-RR using the Co_3_O_4_/Ti cathode (Pt anode; reaction solution, 150 ml; current density, 15 mA/cm^2^; initial pH, 7.0; reaction time, 2 h): **(A)** and **(B)** Rate of loss of NO_3_^−^-N and the fit of the data to first-order kinetics; **(C)** generation of NH_4_^+^-N; and **(D)** product distributions.

The effects of initial NO_3_^−^N concentration on its removal efficiency using the Co_3_O_4_/Ti cathode and the generation of reduction products are shown in Figure 8. At initial NO_3_^−^-N concentrations of <100 mg/L, the removal efficiency of the system was close to 100% at 2 h; and the corresponding reduction products were NH_4_^+^-N (60%) and N_2_. At an initial NO_3_^−^-N concentration of 200 mg/L, the removal efficiency decreased to ∼58%, while the NH_4_^+^-N generation efficiency increased to ∼79%. Under this condition, the higher initial NO_3_^−^-N concentration suppressed the competing hydrogen evolution reaction, thus reducing its charge consumption at the electrode.

### 3.3 Effects of Cl^−^

The main product of electrocatalytic NO_3_^−^ reduction is NH_4_^+^, which is also a contaminant requiring removal. However, in the presence of Cl^−^, which is widely present in drinking water and industrial water, the active species participating in the oxidative transformation of NH_4_^+^-N to N_2_ at the anode (Eqs 6–9) will increase TN removal. The IrO_2_-RuO_2_/Ti electrode is widely employed in the chlor-alkali industry because of its high chlorine evolution performance. To investigate the effects of Cl^−^ on NH_4_^+^-N generation and NO_3_^−^-N removal, various concentrations of Cl^−^ were presented to a Co_3_O_4_/Ti/IrO_2_-RuO_2_/Ti NO_3_^−^-N removal system (Table 1). As the concentration of Cl^−^ increased from 0 to 2000 mg/L, the removal efficiencies of NO_3_^−^-N were all >90%. At 4,000 mg/L Cl^−^, the removal efficiency decreased to 83.99% due to the oxidation of NH_4_^+^ to NO_3_^−^ by HClO/ClO^−^. The increase in Cl^−^ concentration increased the amount of HClO/ClO^−^ generated by anodic oxidation to reduce NH_4_^+^-N to N_2_. Hence, NH_4_^+^-N generation decreased and TN removal efficiency increased with increasing Cl^−^ concentration. The TN removal efficiency reached 78.1% with negligible NH_4_^+^-N generation (0.34%) and without NO_2_^−^-N accumulation.

**TABLE 1:** Effects of Cl^−^ on the Co_3_O_4_ **/**Ti/IrO_2_-RuO_2_/Ti NO_3_^−^-N removal system.

Cl^−^ Concentration (mg/L)	NO_3_^−^-N removal (%)	NO_2_^−^-N generation (%)	NH_4_^+^-N generation (%)	TN removal (%)
0	92.2	—	54.5	24.8
1000	90.7	—	37.2	38.3
2000	91.1	—	19.9	60.3
4,000	84.0	—	0.340	78.1

### 3.4 Long-Term Stability

**FIGURE 9:**
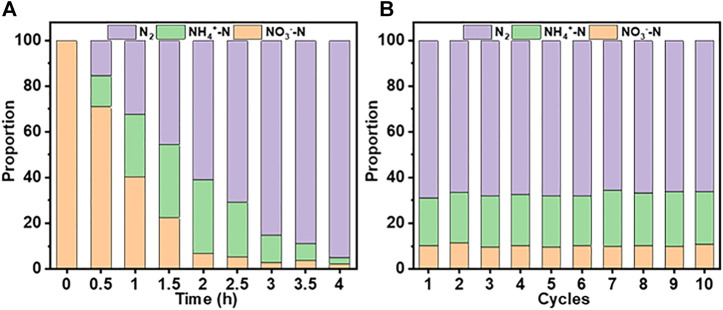
Changes in NO_3_^−^-N removal and the distribution of generated products for the Co_3_O_4_/Ti/IrO_2_-RuO_2_/Ti system ([NO_3_^−^-N], 100 mg/L, 150 ml; current density, 15 mA/cm^2^; pH, 7.0; cycle time, 2 h). **(A)** Changes over 4 h. **(B)** Changes over 10 cycles.

In addition to the initial activity, the long-term performance of a catalyst is an essential requirement for its commercial application. Figure 9 shows the changes in NO_3_^−^-N removal and distribution of generated products over 4 h, and 10 consecutive cycles of 2 h each, using the system. At an initial concentration of 100 mg/L, almost all NO_3_^−^-N is converted into N_2_ after 4 h (Figure 9A). After 10 cycles (Figure 9B), the removal efficiencies of NO_3_^−^-N (∼90%) and TN remained unchanged.

### 3.5 EC and CE

**FIGURE 10:**
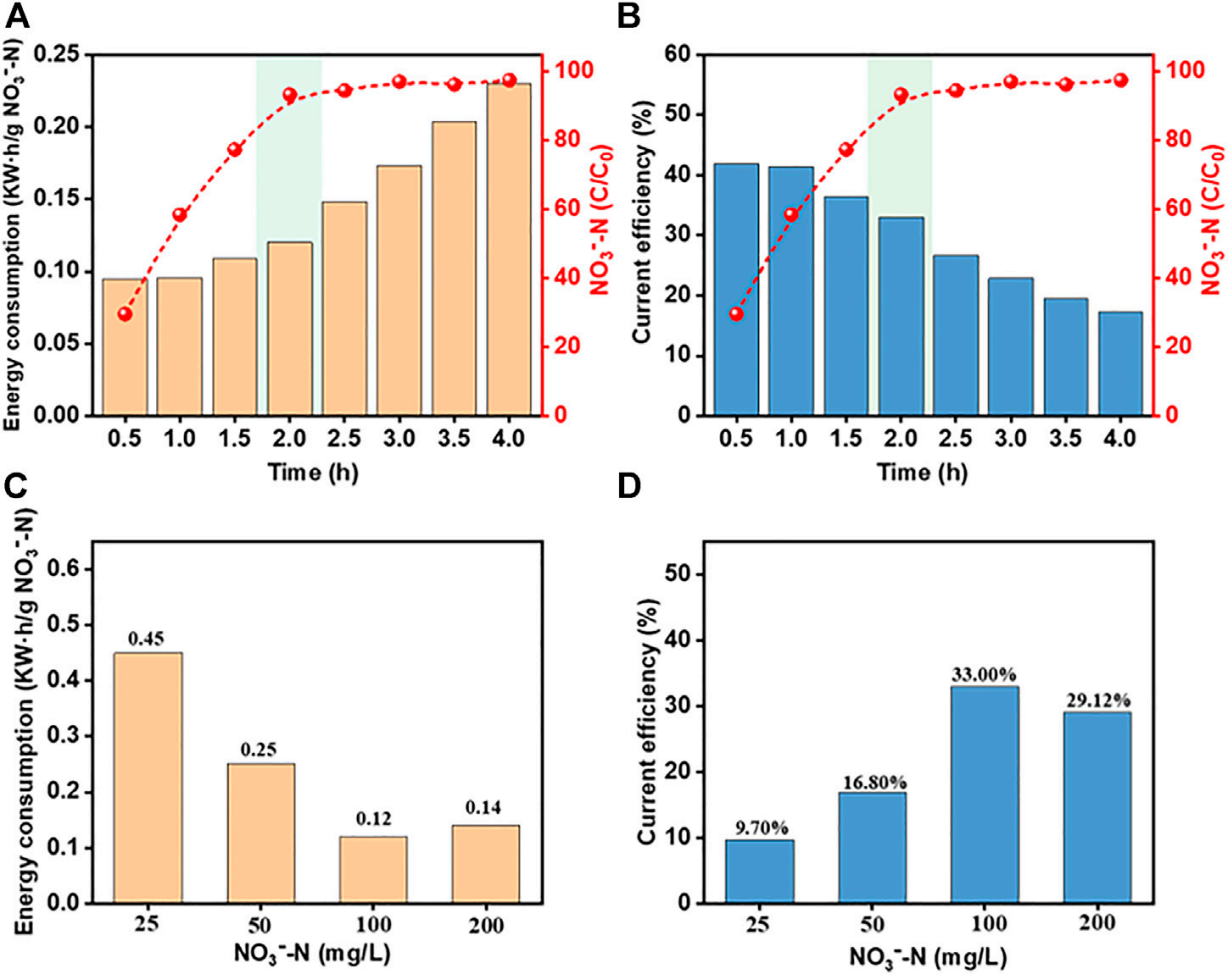
Effects of reaction time and initial NO_3_^−^-N concentrations on the operational efficiency. **(A)** and **(C)** EC; **(B)** and **(D)** CE (pH, 7.0; current density, 15 mA/cm^2^; [Cl^−^], 2000 mg/L; reaction time, 2 h).

EC and CE are key evaluation factors for the commercial electrochemical treatment process (Zeng et al., 2020). The EC and CE under different process conditions using the system were calculated. It can be seen from Figure 10A,B that within 1 h after the start of the reaction, EC is lower and CE is higher than those of the follow-up experiments, but the NO_3_^−^-N removal rate is only 58.52%. After 2 h, the NO_3_^−^-N removal efficiency reaches 93.39% with an EC of 0.10 kW h/g NO_3_^−^-N and a CE of 40.3%. There was no significant improvement in the follow-up, but the EC continued to rise, and the CE continued to decline.

The effect of the initial NO_3_^−^-N concentration is demonstrated in Figures 10C,D. As the NO_3_^−^-N concentration increased, the EC decreased and CE increased. This can be explained by the increase in the contact area between NO_3_^−^-N and the electrode surface with increasing concentrations, which promotes the reduction reaction. From an economic viewpoint, the results indicate that the Co_3_O_4_/Ti/IrO_2_-RuO_2_/Ti electrocatalytic process is more suitable for wastewater with high concentrations of NO_3_^−^. The small amount of NO_3_^−^ remaining in the electrochemically treated wastewater can be removed by other processes, such as the electrocatalytic removal of NO_3_^−^-N, which can be combined with constructed wetlands for wastewater control/remediation.

## 4 Conclusion

A Co_3_O_4_/Ti electrode was successfully prepared by electrodeposition, and the material showed good electrocatalytic performance toward NO_3_^−^-RR. At an initial concentration of 100 mg/L NO_3_^−^-N, the removal rate was ∼98% in 2 h (Pt anode; pH, 7.0; current density, 15 mA/cm^2^). The corresponding generation of NH_4_^+^-N was ∼60%, while NO_2_^−^-N was not detected. When IrO_2_-RuO_2_/Ti was employed as the anode in the presence of Cl^−^ (2000 mg/L), the removal efficiencies for NO_3_^−^-N and TN under the same operating conditions were ∼91% and ∼60%, respectively, with an EC of 0.10 kW h/g NO_3_^−^-N and a CE of 40.3%. After 4 h of continuous operation, 100% of NO_3_^−^-N was converted into N_2_. In addition, the system could maintain the removal efficiencies of ∼90% and ∼60% for NO_3_^−^-N and TN, respectively, after 10 consecutive cycles (2 h each). This work provides a simple preparation method of electrodeposited Co_3_O_4_/Ti with good catalytic performance and stability, which provides a new preparation strategy for the Co_3_O_4_ catalytic electrode.

## Data Availability

The original contributions presented in the study are included in the article/Supplementary Material, further inquiries can be directed to the corresponding author.
